# Exploiting the Achilles’ heel of membrane trafficking in trypanosomes

**DOI:** 10.1016/j.mib.2016.08.005

**Published:** 2016-12

**Authors:** Martin Zoltner, David Horn, Harry P de Koning, Mark C Field

**Affiliations:** 1 School of Life Sciences, University of Dundee, Dundee DD1 5EH, Scotland, UK; 2 Institute of Infection, Immunity and Inflammation, University of Glasgow, Glasgow G12 8TA, Scotland, UK

## Abstract

Pathogenic protozoa are evolutionarily highly divergent from their metazoan hosts, reflected in many aspects of their biology. One particularly important parasite taxon is the trypanosomatids. Multiple transmission modes, distinct life cycles and exploitation of many host species attests to great prowess as parasites, and adaptability for efficient, chronic infection. Genome sequencing has begun uncovering how trypanosomatids are well suited to parasitism, and recent genetic screening and cell biology are revealing new aspects of how to control these organisms and prevent disease. Importantly, several lines of evidence suggest that membrane transport processes are central for the sensitivity towards several frontline drugs.

## Highlights

• Development of new drugs against trypanosomes is a crucial but unmet need.
• Membrane transport, endocytosis and related processes have been proposed as drug targets.
• Recent insights uncovered the mode of action of two drugs that are already in the clinic.
• Both of these drugs, suramin and pentamidine, bind surface proteins.
• It is possible that endocytosis is a common component of sensitivity to suramin and pentamidine.

**Current Opinion in Microbiology** 2016, **34**:97–103

This review comes from a themed issue on **Parasitic and fungal diseases**

Edited by **Gero Steinberg**

For a complete overview see the Issue and the Editorial

Available online 9th September 2016

**http://dx.doi.org/10.1016/j.mib.2016.08.005**

1369-5274/© 2016 The Authors. Published by Elsevier Ltd. This is an open access article under the CC BY license (http://creativecommons.org/licenses/by/4.0/).

## African trypanosomes: novelty and conservation

Trypanosomatids cause a very broad range of diseases afflicting humans, animals, livestock, fish, plants and wild animals. Evidence has emerged for preadaptation to parasitism during evolution of the group and ongoing genetic modifications to suit distinct modes of infection and immune evasion [1••, 2••]. Trypanosomes are classified as Excavata, and branched early from the eukaryotic lineage [3]. This fuelled considerable optimism that when the genomes of these organisms were characterized, a wealth of drug and potential therapeutic targets would emerge. Approximately forty percent of trypanosome protein-coding genes appear either lineage-specific or of such great divergence that they present a viable target, despite having an ortholog in higher eukaryotes [4, 5]. This promise has, however, failed to emerge for several reasons, not least of which is translating initial hit compound activity against specific protein targets to leads with promising activity against whole cells, that is, trypanocidal or trypanostatic activity. Consequently, many efforts identifying new drugs remain focused on classical approaches such as phenotypic screens and do not, at least *a priori*, engage with either genetic divergence or those cell biological aspects unique to the kinetoplastids [6^•^]. Even compounds emerging from various screening efforts, with promising *in vitro* activity have experienced low rates of translation into viable (pre)-clinical candidates. However, serendipitously, it has emerged that many drugs presently used against these parasites, and specifically the African trypanosomes, do target rather well known unique aspects of trypanosome cell biology, and/or require these features, for their specificity and high potency.

The trypanosome cell is elongated, with a morphology supported by a sophisticated and elaborate subpellicular microtubule array [7, 8]. This feature essentially precludes budding of conventional transport vesicles from the vast majority of the plasma membrane, and all membrane flow to and from the surface is restricted to the flagellar pocket. Thus, drugs that do not effectively diffuse across membranes, must reach their intracellular targets via the flagellar pocket or cross the membrane via an alternate mechanism, that is, a transporter or channel. The flagellar pocket crucially is devoid of the microtubule array, and while the membrane is continuous with the bulk plasma membrane, it has a distinct protein and lipid composition and is physically delineated by a complex collar surrounding the pocket neck, which likely also restricts fluid phase diffusion [8, 9].

Trypanosomes possess a conventional endomembrane system, including a Golgi complex, early and recycling endosomes, late endosomes incorporating the ESCRT/multi-vesicular body system and a terminal endosome or lysosome, albeit somewhat streamlined, with several of these organelles probably present in interphase cells as single copy [10, 11]. Several features are highly unique, for example, the mammalian-infective form of *T. brucei* has an extreme rate of endocytosis, capable of turning over the plasma membrane many times per hour, contributing towards removal of surface-bound immune effectors, aiding immune evasion [12^•^]. The surface is dominated by the variant surface glycoprotein (VSG), a GPI-anchored homodimer comprising 90% of surface protein. Other surface proteins possess *trans*-membrane domains, but, importantly, are often highly divergent and trypanosome-specific [13•, 14].

Trypanosome endocytosis is exclusively clathrin-dependent, setting it apart from higher eukaryotes where multiple modes of endocytosis operate [15]. Furthermore, the widely conserved heterotetrameric (AP)-2 adaptin complex is absent and is inversely correlated with the presence of VSG and thus antigenic variation, the principal mechanism of immune evasion [16, 12•]. Additional clathrin adaptor proteins are present and include both ENTH and ANTH-domain phosphoinositide-binding proteins [17] and a cohort of trypanosomatid-specific proteins [18]. Sorting surface *trans*-membrane proteins requires ubiquitylation, likely performed by divergent ubiquitin ligases, although these remain unidentified [19^••^]. Whilst these details indicate a distinct endocytic system, the level of conservation with, for example, *Saccharomyces cerevisiae*, is considerable and the divergence certainly appears less extreme than in Apicomplexan parasites, where entire compartments and pathways have been repurposed [20, 21•].

Membrane transport activity is rather different between life stages and species of parasite, which may in part explain the differential sensitivity to some front-line drugs. Specifically, the bloodstream forms of *T. brucei* have much greater endocytic transport rates compared with the insect forms, and this correlates with sensitivity to suramin and pentamidine for example, with procyclic cells being much less sensitive to either drug, although other changes to the surface composition are considerable and may also contribute to the differential sensitivity. Although pentamidine is almost exclusively used against *T. brucei gambiense*, the West African form of the disease, and suramin usually for the East African *T. brucei rhodesiense*, both subspecies are fully sensitive to either drug. Both drugs are used exclusively to treat early stage (haemolymphatic) trypanosomiasis, because neither drug penetrates sufficiently into the central nervous system; it is not known how trypanosomes adapt after crossing the blood–brain barrier, and whether this might impact drug sensitivity.

In addition to endocytic mechanisms for entry into the cell, trypanosomes also possess a considerable array of surface nucleoside and nucleobase transporters, together with hexose and amino acid permeases, plus a small family of aquaporins [22]. These systems obviously also present a potential mechanism for accumulation of drugs as well as natural metabolites, whilst themselves also being proteins that are subject to turnover by the endocytic system. By contrast to many surface proteins, the transporters appear to be more broadly conserved with higher eukaryotes. Significantly, traffic focused at the flagellar pocket, high rates of endocytosis, novel surface protein composition and the presence of conserved transporters all directly contribute towards sensitivity of African trypanosomes to drugs that have been in clinical use since the 1920s.

## A grandfather therapeutic: suramin

**Figure 1:**
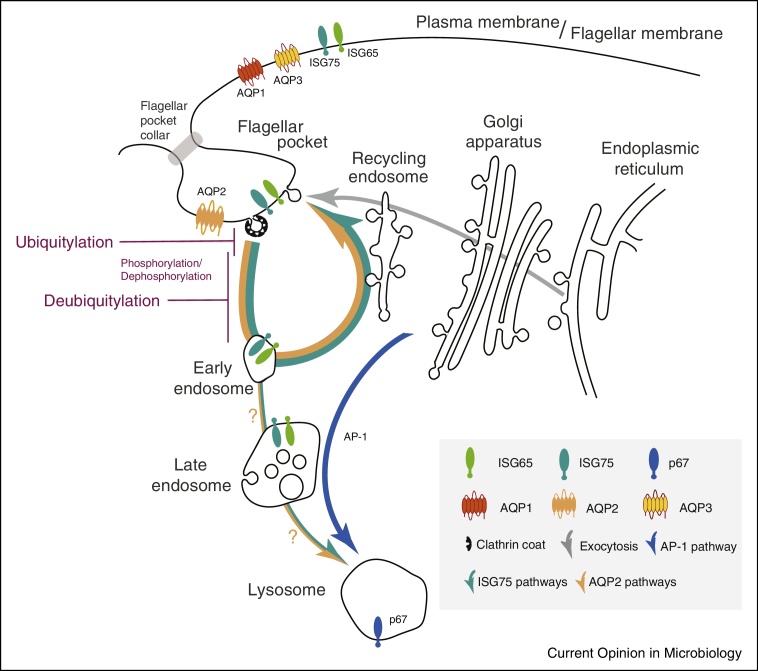
The trypanosome endosomal system. A simplified schematic of the trypanosome endomembrane system is shown, with the flagellar pocket at top left. Teal and orange arrows indicate degradative and recycling trafficking routes, blue putative AP-1-mediated transport from the Golgi complex to the lysosome and gray exocytic/biosynthetic pathways. The predominant locations of ISG75, ISG65, aquaglyceroporins and p67 (the major lysosomal protein) are indicated by icons. Evidence suggests that ISG75 is ubiquitylated at, or close to the surface (magenta) and deubiquitylation by TbUsp7 and/or TbVdu1 is proposed to take place before the sorting step at the early endosome that selects for the recycling or degradative arm of the endocytic system. TbVdu1 is known to associate with structures in this region, whilst TbUsp7 is likely cytosolic. AQP2 localization is restricted to the flagellar pocket, while AQP1 and AQP3 are predominantly on the flagellar membrane and plasma membrane, respectively. AQP2 has been recently described as high-affinity pentamidine receptor and this raises the possibility of endocytotic uptake.

**Figure 2:**
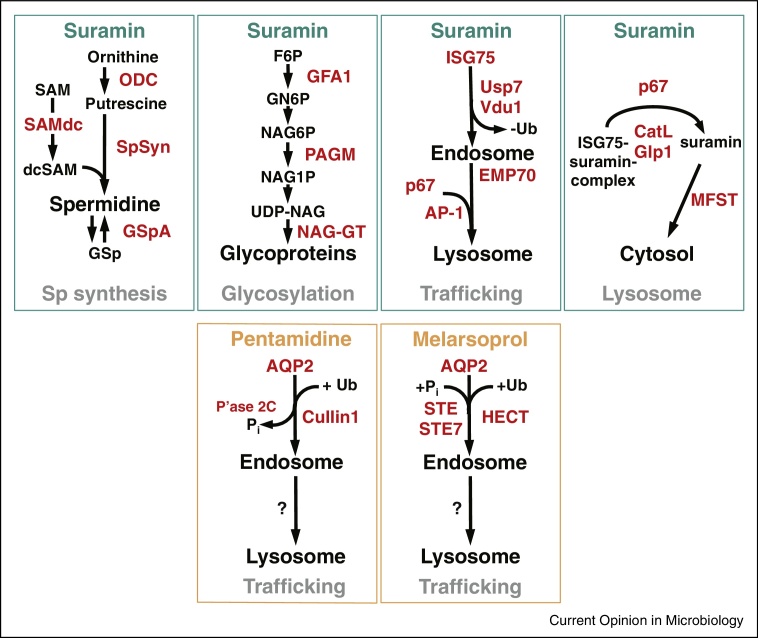
Mode of action of trypanocidal drugs. Summary of biochemical pathways linked to drug action for suramin, pentamidine and melarsoprol. Proteins sensitizing to the respective drug, as identified in a genome-wide loss-of-function screen [26^••^] are drawn in red. (Pi: inorganic phosphate, Ub: ubiquitin).

Suramin emerged from early synthetic chemistry and development of aniline dyes [23]. The trypanocidal diazo dyes, trypan red and trypan blue, were developed by Bayer in 1916 and led to suramin, still a frontline treatment against some forms of trypanosomiasis [24]. High molecular weight and negative charge prevent passive membrane diffusion, suggesting specific uptake. A thousand-fold reduced potency against insect stage parasites suggested involvement of endocytosis, as endocytic trafficking is much decreased in this life stage [25]. However, extensive surface proteome remodelling between life stages also suggests the possibility of bloodstream stage-specific expression of a ‘suramin-receptor’ [14]. Genome-wide loss-of-function screens identified multiple genes that sensitize trypanosomes to suramin [26^••^] (Figure 2), many of which have roles and/or locations at the endocytic pathway, for example, invariant surface glycoprotein 75 (ISG75), two deubiquitylating enzymes (DUBs) Usp7 and Vdu1, the AP-1 adaptin complex and the lysosomal protein p67 [27]. An ISG75-dependent pathway is required for lysosomal delivery of suramin while an AP-1-dependent path is likely connected to lysosomal composition and a requirement for transport of p67 and other factors (Figure 1) [19^••^]. ISG75 stability is regulated by ubiquitylation [28] and evidence that trypanosome Usp7 and Vdu1 modulate ISG75 turnover is consistent with this model [19^••^]. Significantly, the suramin-uptake pathway is highly specific and does not involve the closely related invariant surface glycoprotein 65 (ISG65) family.

Together these observations are consistent with a hypothesis that ISG75 is the suramin receptor, but failure to demonstrate binding *in vitro* to recombinant ISG75 (unpublished data, MZ, MCF) suggests that additional factors may be involved. Suramin binds various serum proteins with high affinity, including Low Density Lipoprotein (LDL), and the influence of LDL on suramin uptake has led to a proposed model of an LDL-dependent receptor-mediated pathway for suramin internalization [29]. However, this was overturned by the mutation of trafficking pathway components [25]. Formal proof of receptor identity and precise mechanisms for suramin uptake remain elusive, but what is clear is the essential role for endocytosis and a protein with an itinerary that includes transport through the endosomal system. Significantly, the lysosomal proteases CatL and CBP1 are also implicated for suramin-sensitivity, and potentially these proteases are required for degradation of ISG75 to release suramin into the lysosome [30]. Notably, recent evidence indicates that CatL also degrades human serum trypanolytic factors and that a lysosomal inhibitor of a cysteine peptidase, modulates this protease activity [31^•^].

Despite these advances the mechanism of suramin toxicity remains elusive. Suramin exhibits considerable polypharmacology and can bind and inhibit a wide variety of distinct protein families (see e.g. [32, 33, 34]). Although suramin inhibits all seven trypanosome glycolytic enzymes plus cytosolic pyruvate kinase (PYK) in the micromolar range [35], it inhibits bloodstream stage trypanosome proliferation at nanomolar concentrations [26^••^], making it unlikely that glycolysis represents the primary target. However, endocytosis may accumulate the drug to higher intracellular levels than the external concentration; importantly the highly charged suramin molecule, once internalized, cannot easily escape from the cell. Again, this represents a remarkable impact from unique aspects of trypanosome cell biology in enabling specific toxicity, and is currently beyond the ability of rational drug design to predict or replicate. However, these features do indicate potential cellular aspects that are exploitable.

## Old and still in the clinic: pentamidine and melarsoprol

Aromatic diamidine drugs including pentamidine, and organic arsenicals like melarsoprol, are highly cytotoxic to most cells they enter. Therefore toxicity is predominantly determined by uptake across the plasma membrane, and selectivity rests on the expression of efficient cell-surface transporters by the parasite. Conversely, mutations in such transporters diminishing transport rates or loss of substrate recognition can lead to drug resistance [36]. Pentamidine and melarsoprol, despite very different structures, exhibit cross-resistance in *Trypanosoma brucei* [37]. Since pentamidine and melarsoprol have distinct cellular targets, cross resistance may reside within the uptake mechanism. An unusual aminopurine transporter, TbAT1/P2, a member of the highly conserved Equilibrative Nucleoside Transporter family, facilitates uptake of both [37]. Deletion or mutation of the TbAT1/P2 gene reduces the sensitivity of *T. brucei* to diamidines, particularly diminazene, and melaminophenyl arsenicals [38, 39, 40, 41, 42].

Significantly, loss of TbAT1/P2 alone does not recreate the high levels of melarsoprol-pentamidine cross-resistance (MPXR) observed [39, 43]. *T. brucei* expresses additional transport systems for pentamidine and a genome wide RNAi screen identified the *TbAQP2/AQP3* locus as the genetic determinant for MPXR [26••, 44•]. Further analyses revealed that aquaglyceroporin 2 (*AQP2*) determines MPXR [44•, 45•]. Further, re-introduction of a wild-type *TbAQP2* allele in even the most resistant strains fully restores drug sensitivity [45•, 46••, 47•, 41]. Indeed, expressing *TbAQP2* in *Leishmania mexicana* promastigotes profoundly increases their sensitivity to both pentamidine and melarsoprol [45^•^].

However, the translocation mechanism of TbAQP2 is less obvious as aquaglyceroporins are not known to allow passage of molecules of high molecular weight like melarsoprol or pentamidine. TbAQP2 does have a unique selectivity filter; several large, aromatic amino acids that normally restrict the pore are replaced by smaller side chain residues, and a key arginine is replaced with leucine [44^•^]. These changes are expected to enlarge the pore size and thus allow passage of cations, including the highly flexible pentamidine molecule [36]. However, an alternative model, in which AQP2 binds pentamidine and mediates internalization via receptor-mediated endocytosis, was recently proposed [48^••^]. This is an attractive alternative, marrying the implausibility of large-molecule transport by TbAQP2, an unusually high affinity for pentamidine, the high endocytosis of bloodstream trypanosomes [12^•^] and localization of TbAQP2 to the flagellar pocket [44^•^]. The flagellar pocket localization is consistent with uptake by endocytosis (Figure 1). Thus, uptake of both suramin and pentamidine, and potentially even melarsoprol, may depend upon their affinities for surface receptors, with subsequent membrane trafficking (Figure 2).

The genes identified through genome-wide screens [26^••^] for pentamidine/melarsoprol and suramin sensitivity do feature a significant overlap but this does not implicate common endocytic components. However, additional evidence from these screens is consistent with involvement of trafficking factors downstream of AQP2 [26^••^]. Indeed, for both pentamidine and melarsoprol, the screens identified proteins likely involved in phosphorylation (Phosphatase 2C) and ubiquitylation (Cullin1) (Figure 2), post-translational modifications implicated in regulating endocytosis of mammalian AQP [49]. This is reminiscent of the DUBs that sensitize trypanosomes to suramin and regulate ISG75 [19^••^]. However, if these proteins are involved in the promotion of AQP2 internalization and turnover, knockdown could change pentamidine sensitivity regardless of whether the drug binds the channel or translocates through it. Finally, the endocytosis model does not explain all previous observations on pentamidine uptake in *T. brucei*, such as a 10-fold higher rate of high-affinity pentamidine uptake in procyclic forms than in bloodstream forms [50] despite TbAQP2 distribution across the entire procyclic cell surface [44^•^], or the high uptake rate when TbAQP2 is expressed in *Leishmania* promastigotes [45^•^]. Both procyclic and promastigotes have much lower endocytosis than the bloodstream *T. brucei* [25, 51], and further study is essential to settle the mechanism of TbAQP2-mediated drug transport.

## Perspectives

Serendipity will always be an important component of discovery, and drug uptake is a good exemplar. Recognising the specific binding properties of aniline dyes to fabrics in the early years of synthetic chemistry was rightly interpreted as representing specificity, and ultimately evolved into Ehrlich's concept of the ‘magic bullet’ that kills a pathogen but not the host. Ehrlich also reported on the activities of trypan blue and trypan red, two trypanocidal compounds leading to the development of suramin. The unique features of the flagellar pocket and high endocytic rate have long been considered as potential routes to therapy and demonstrations that innate immune mechanisms also rely on endocytic trafficking has strengthened this [52]. Many experimental interventions compromising endocytic trafficking lead to the rapid death of African trypanosomes [53], and it is rather a curious realisation that the properties of this system were being exploited by highly effective therapeutic agents developed almost a century ago. There is much scope for exploiting these drug uptake mechanisms to deliver new drugs, especially given the surprising variety of compounds that they internalize. Notably, drug-loaded nanoparticles, coated with a nanobody specifically targeting the trypanosome surface, can be used to bypass the usual delivery route altogether and deliver drugs via endocytosis [47^•^]. Indeed, this approach actually increases the efficacy of pentamidine, and a similar approach is likely to be effective for delivering other drugs. Harnessing these features represents attractive and tractable opportunities, as does direct screening for inhibitors of the novel components that control trafficking and surface protein transport. Finally, there are at least two components required for trypanocidal activity, entry and activity against a target; rather unexpectedly it is emerging that it is the former that may be more crucial for specificity, at least for current drugs.

## References and recommended reading

Papers of particular interest, published within the period of review, have been highlighted as:

• of special interest
•• of outstanding interest
